# GIS-Based Forest Fire Susceptibility Zonation with IoT Sensor Network Support, Case Study—Nature Park Golija, Serbia

**DOI:** 10.3390/s21196520

**Published:** 2021-09-29

**Authors:** Ivan Novkovic, Goran B. Markovic, Djordje Lukic, Slavoljub Dragicevic, Marko Milosevic, Snezana Djurdjic, Ivan Samardzic, Tijana Lezaic, Marija Tadic

**Affiliations:** 1 Faculty of Geography, University of Belgrade, Studentski trg 3/III, 11000 Belgrade, Serbia; ivan.novkovic@gef.bg.ac.rs (I.N.); slavoljub.dragicevic@gef.bg.ac.rs (S.D.); milosevic.marko7@yahoo.com (M.M.); snezana.djurdjic@gef.bg.ac.rs (S.D.); ivan.samardzic@gef.bg.ac.rs (I.S.); tijana.lezaic@gef.bg.ac.rs (T.L.); 2 School of Electrical Engineering, University of Belgrade, Bulevar Kralja Aleksandra 73, 11120 Belgrade, Serbia; 3 Aspire Technology Unlimited, Vladimira Popovica 6, Unit B10, 11070 Belgrade, Serbia; djordje.lukic@aspiretechnology.com; 4 Klett Publishing House, Maršala Birjuzova 3–5, 11000 Belgrade, Serbia; makitadic11@gmail.com

**Keywords:** forest fire susceptibility, GIS, remote sensing, random forest (RF), IoT sensor networks, fire outbreak occurrence, fuzzy analytic hierarchy process (fuzzy AHP), technique for order of preference by similarity to ideal solution (TOPSIS)

## Abstract

The territory of the Republic of Serbia is vulnerable to various natural disasters, among which forest fires stand out. In relation with climate changes, the number of forest fires in Serbia has been increasing from year to year. Protected natural areas are especially endangered by wildfires. For Nature Park Golija, as the second largest in Serbia, with an area of 75,183 ha, and with MaB Reserve Golija-Studenica on part of its territory (53,804 ha), more attention should be paid in terms of forest fire mitigation. GIS and multi-criteria decision analysis are indispensable when it comes to spatial analysis for the purpose of natural disaster risk management. Index-based and fuzzy AHP methods were used, together with TOPSIS method for forest fire susceptibility zonation. Very high and high forest fire susceptibility zone were recorded on 26.85% (Forest Fire Susceptibility Index) and 25.75% (fuzzy AHP). The additional support for forest fire prevention is realized through an additional Internet of Thing (IoT)-based sensor network that enables the continuous collection of local meteorological and environmental data, which enables low-cost and reliable real-time fire risk assessment and detection and the improved long-term and short-term forest fire susceptibility assessment. Obtained results can be applied for adequate forest fire risk management, improvement of the monitoring, and early warning systems in the Republic of Serbia, but are also important for relevant authorities at national, regional, and local level, which will be able to coordinate and intervene in a case of emergency events.

## 1. Introduction

The territory of the Republic of Serbia is the most vulnerable by earthquakes, landslides, riverine and torrential floods, hailstorms, droughts, and forest fires, which are the most frequent and the most destructive natural disasters regarding huge material damage and loss of human lives. In the last few decades, upward trends in both the number of forest fire events and burned areas have been confirmed in Europe. In relation with climate changes, during the high-temperature summer months, the number of forest fires in Serbia has been increasing from year to year. Forest area in the Republic of Serbia covers 27,200 km^2^, which is approximately 31.1% of the country area [1]. During the period from 1990 to 2005, around 43,000 ha of Serbian forest and overgrown forest soil were ruined as a consequence of forest fires. Within this period, the largest number of fires was registered in 2000, as 13,201 ha of forest and overgrown forest soil was burnt in 339 fires [2].

Analyzing the number of forest fires, Lukić et al. [3] found that the number of forest fires increased in Serbia during the period from 2000 to 2012, but this was non-significant. The largest number of fires (28,546) happened in 2007 in Serbia, when the highest air temperature was recorded on July 24th in the meteorological station Smederevska Palanka (temperature of 44.9 °C). The year 2012 saw the longest heatwaves during the summer and the worst drought since the beginning of observations in Serbia. More than 1000 forest fires occurred during that time. According to Unkašević and Tošić [4,5], the last few decades in Europe have been characterized by the steepest temperature increase since the beginning of the twentieth century. Vuković et al. [6] predicted that in the near future (2016–2035), mean temperature in Serbia is expected to increase by 1 °C on average, with larger differences in summer than in autumn. Based on that prediction, an increase in the number and intensity of forest fires is expected.

In accordance with the results of previous research and future climate change scenarios, the topic of research is of great importance, especially in the context of improving protection of nature in the most sensitive ecological areas. Protected natural areas are particularly vulnerable to forest fires. The emergence of this natural disaster can cause irreversible damage to those sensitive ecosystems and even cause the extinction of certain species. In the recent past, examples of fires in protected natural areas in Serbia have not been so uncommon. According to the most accurate data, 7.66% or 677.950 ha of the total surface area of Serbia is under legal protection, which is based on the Law of Nature Protection [7]. The Nature Park Golija is the second largest protected area in the country, and it covers about 11% of the total protected areas in Serbia. The fact that it is one of largest protected areas in Serbia, known for its geodiversity and biodiversity, and also that the part of this protected area is a UNESCO MaB reserve, emphasizes its importance for nature conservation. Unfortunately, this area has not been spared by the occurrence of wildfires in recent years.

The vulnerability of the forest area to a fire is dependent on diverse factors such as vegetation, topography, and distance from roads, rivers, and human settlements [8], beyond the weather. The objective of this paper is to determine zones of different forest fire susceptibility using Geographic Information System (GIS) and multi-criteria decision analysis (MCDA) in order to enable adequate forest fire risk management in protected natural area such as Nature Park Golija. The use of GIS is unavoidable today when it comes to spatial-related research. This is of utmost importance in order to achieve appropriate understanding of environment and the processes that take place in it. One of the key processes of MCDA is its ability to combine the values of multiple criteria with a single evaluation score for each alternative decision [9]. Thus, the integration of MDCA methods in the spatial domain provides a proper framework for fire risk assessment [10].

In order to identify the regions susceptible to fire and determine forest fire risk, various studies have been carried out worldwide. Among many methods used for modelling of forest fire susceptibility are index-based method [11,12], fuzzy analytic hierarchy process (AHP) [9,10,13,14], and fuzzy analytic network process (ANP) [15]. Since conventional AHP technique might not be adequate to provide effective decision in forest fire susceptibility mapping, fuzzy AHP technique has been used to address this issue by enabling decision makers to define approximate preferences through fuzzy numbers. This gives a much better and more precise representation of the relationship between criteria and alternatives [16].

The most significant contribution of this research is that the fuzzy AHP method was used for the first time for forest fire susceptibility assessment in Serbia. The fact that it was used on the territory of one of the most important protected natural areas reinforces that significance. Additionally, it compares results of index-based methods and fuzzy AHP using the technique for order of preference by similarity to ideal solution (TOPSIS) method. No less important is the fact that the data and software (QGIS, RStudio) used for forest fire susceptibility zonation is completely open source. Zero cost provided in such a way should help better forest fire risk management for communities that have a low budget when it comes to nature protection. Although it is well known that historical data of forest fires that occurred in certain areas and relevant meteorological data are important for comprehensive forest fire risk assessment, this paper shows that it is possible to perform suitable forest fire susceptibility zonation even if such datasets are not available. Another contribution of this paper is the improvement of classification of land use/land cover/vegetation based on the remote sensing data and the improvement of the random forest (RF) method in such a classification.

As stated, in this study, we deployed the fuzzy AHP method without historical data of forest fires that occurred in a given area (Nature Park Golija) and relevant meteorological data used as inputs. In some previous studies [14,15,17,18,19,20], meteorological data, marked also as climate or weather data, were used as inputs to fuzzy AHP methods, along with other parameters (i.e., topographic, vegetation, human), in order to assess forest fire susceptibility. It can be argued that by using local meteorological data, i.e., data collected by the sensors densely deployed in area of interest, we could further improve quality of the forest fire susceptibility assessment in a long-term manner (i.e., monthly or yearly by using averaged data) or in a short-term manner (i.e., daily by using current data). Such an approach would also allow us to gather and use more precise information regarding the influence of global climate changes on local climate parameters (for the given area), which cannot be ignored in the current situation. In the case of protected natural areas (such as NP Golija), this improved insight could be beneficial not only for forest fire prevention, but also for wildlife preservation. Additionally, the inherent real-time nature of the sensor network operation enables us to observe other possible applications in forest fire prevention, detection, and monitoring such as real-time forest fire risk assessment and detection. Thus, as an addition, we here present a general framework for Internet of Thing (IoT)-based sensor networks that could be established in the given area of Nature Park Golija in order to enable continuous collection of local meteorological data. This general solution is here designed based on the comprehensive overview and analysis of the previously suggested solutions and communications technologies for this purpose. The general description of the proposed IoT-based sensor network is given in Section 4, as well as the discussion regarding the choice of its elements (i.e., communications architecture, set of weather and environmental parameters used for forest fire detection and fire risk assessment, etc.), while the adopted fuzzy AHP inference methods used for the real-time fire risk assessment and detection, as well as for the improved the forest fire susceptibility assessment, are briefly presented in Section 2.4.

The main objectives of this research are 1) creation of forest fire susceptibility maps with emphasized zones of very high and high susceptibility, 2) designing a proper sensor network in order to improve early warning systems, and 3) obtaining adequate spatial data that will help forest fire mitigation and risk management.

### Study Area

The study area of this research is Nature Park Golija established in 2001, which is situated in the south-western part of Serbia. The core area and largest part of this nature park is Mt. Golija, with its highest peak Jankov kamen at 1833 m above sea level. It is characterized by dynamic relief dissection with various geomorphological objects and phenomena, which are spreading through a 32 km-long mountain region into altitude zones from 416 m to 1833 m. The major mountain ridge is spreading in the north–south direction from the highest mountain peak to the Valley of the Golijska Moravica river at 620 m. As a result of the complex composition of geological structures, various geosites are placed into the research area, and some of them are of great scientific and educational importance as objects of geoheritage (e.g., Upper Cretaceous rudist limestones geosites) [21].

According to the geographical position of the Nature Park Golija territory (43.233445°–43.55110° N; 20.15034°–20.57040° E) and complex climate zonation in relation with altitude, three climate regions can be differentiated: climate zone up to 700 m with the characteristics of moderate continental climate influenced by the surrounding mountains, modified region (700–1.300 m) with long and severe winters and short and fresh summers, and mountain region (over 1.300 m altitude) with subalpine climate. The average annual air temperature is about 9.5 °C in the region up to 500 m altitude (the real value is slightly lower due to the geographical position of the meteorological stations). Considering the large difference between these altitude belts, the area of about 1000 m altitude has an average annual temperature of about 6.5 °C (Sjenica) and 5 °C (Bele Vode, Golija). The average annual precipitation in the region ranges from 572 to 1.077 mm, and the advantage is that about 55–65% of precipitation is excreted in the vegetation period from April to October. Compared with the average value, the largest fluctuations in precipitation are recorded in October, November, and May [22].

The diversity and specificity of the Nature Park Golija area biotope determines the high diversity of flora, vegetation, fauna, and ecosystems that are characterized by a marked degree of representativeness, autochthonous features, and authenticity of nature. Of the special scientific and ecological importance is vegetation associated with tertiary relict species Greek maple (*Acer heldreichii Orph ex. Boiss.*), which is also endemic for some mountains of the Balkan Peninsula. Forests with Greek maple on Mt. Golija are best developed predominantly on slopes (15–20°) exposed to the north, northeast, and northwest from altitudes between 1400 and 1750 m. Greek maple can be found in all floristic combinations of mixed deciduous–coniferous forests in the study area, although its ecological optimum is mixed deciduous–coniferous forests, and as a species, its optimum is in subalpine beech forests. This fact emphasizes the importance of Golija as refugial habitat, as one of the centers of distribution of Greek maples in the Balkan Peninsula [23].

The vegetation is characterized by zonal distribution caused by changes in abiotic factors (e.g., climate, characteristics of soil, etc.) with increase in altitude. In relation to changes with altitude, there are generally two zones of vegetation: (1) mesophylous beech, mixed beech, and coniferous forests; (2) mountain coniferous forests. At the lowest altitudes of the beech forest belt, prevalence has montane beech forests (*Fagenion moesiacae montanum*) on the various types of acid soils and suballiance of beech fir forests *Abieti-Fagenion moesiacae* on the deep, brown, and moderately acid soils, which cover the mid-montane belt. A particular forest type of European spruce, European fir, and beech (*Piceo, Abieti, Fagetum*) is the most productive forest type and the most complex type with the richest floristic composition on the Mt. Golija. The subalpine beech forests (*Fagenion moesiacae subalpinum*) are characterized by low and much branched habit, with a short growing season on the different soil types on the limestone or siliceous geological base [24]. The highest forest belt is composed of spruce forests (*Piceion excelsae*) on the various mountain soils.

**Figure 1:**
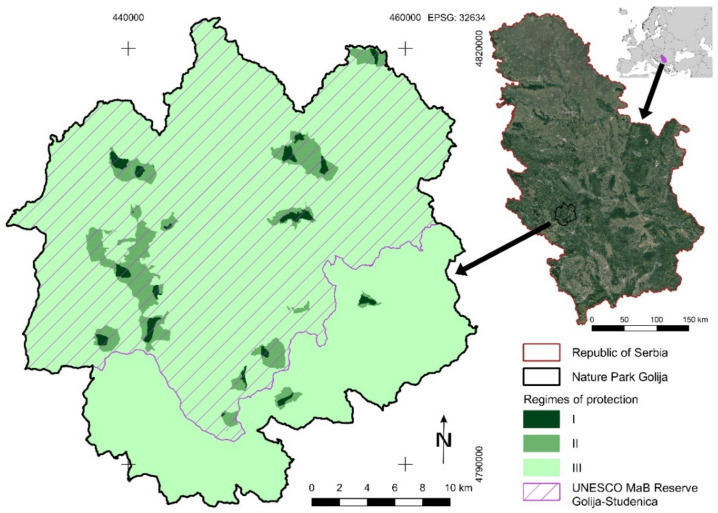
Location of Nature Park Golija and UNESCO Man and Biosphere (MaB) Reserve Golija-Studenica in Serbia Zones of protection regimes in the Nature Park Golija.

The Nature Park Golija covers an area of 75,183 ha comprising the municipalities of Ivanjica, Kraljevo, Raška, Novi Pazar, and Sjenica (36.32% of total area is under state ownership status, and 63.68% is under private ownership status). In order to protect complex natural values of study area, protection regimes have been established [25,26,27]. The area of the most rigorous protection status (first protection regime) occupies the total area of 553.80 ha (it comprises 18 localities). The area of the second protection regime occupies 3883.10 ha (20 localities are protected under this regime). The most liberal protection status (third protection regime) has a major part of Nature Park Golija, or 70,746.10 ha [27,28,29]. The division of Nature Park territory into protection zones is depicted in Figure 1. Previous data are of great importance in the process of managing the natural values and features of the park, especially the ones related to the management of forest fire as a natural disaster or equally as a man-made disaster. Since the zone with the first protection regime is the most important for biodiversity conservation in the protection of natural areas in Serbia, it is necessary to analyze forest fire susceptibility on the protection regime level for the purpose of protection from this disaster.

The impact of the climate and other physical–geographical conditions that may trigger forest fire occurrence is analyzed by Živanović and Vukin [30]. They found that the lowest values of the optimal relief slope for the influx of solar radiation on Golija Mt. are in June (8°) and the highest values are in December and January (61°). The optimal angle for the influx of solar energy throughout the year is 33° to the horizontal surface. The minimum energy is on the slopes oriented toward the north, where the probability of fire occurrence and the uncontrolled spread of fire is lower. Maximum radiation measured on a monthly basis is on the surfaces oriented towards the south during July on the slopes from 10° to 15°, when the fire risk is the highest [30].

The part of the Nature Park Golija along with the Studenica Monastery has been declared a UNESCO Man and Biosphere (MaB) Reserve (53,804 ha) under the name Golija-Studenica in 2001. The UNESCO MaB Programme is aimed towards establishing the functional protection of nature in balance with humans and their social and economic development. The richness of biological communities of Nature Park Golija is recognized in the context of an internationally harmonized approach of nature protection as a program of the important areas for different groups of biotas. The fact that there are only two UNESCO MaB Reserves in Serbia, and that Studenica Monastery is a UNESCO World Heritage Site, gives great importance to this area. The NP Golija is one of the 43 internationally important areas from the territory of Serbia for bird fauna (IBA) and one of the 40 prime butterfly areas (PBA) in Serbia. At the same time, it has international significance as one of the 61 important plant areas (IPA) in the territory of Serbia. Nature Park Golija is also one of the 61 national localities of the Emerald ecological network, and it will be part of the European ecological network Natura 2000 after the accession process of Serbia to the EU.

## 2. Materials and Methods

### 2.1. Forest Fire Susceptibility Index

It is well known that the high probability of the forest fire occurrence is predetermined with a specific state of climatic elements in a certain area, but it is also important to determine where fire would most likely emerge under certain conditions. Therefore, it is necessary to analyze other natural conditions that determine whether a certain territory is more or less susceptible to forest fires, especially the type of vegetation and terrain topography. Additionally, it is important to determine the scope and character of human activities, since man is the main cause of this disaster [31]. Forest fire susceptibility is calculated on the basis of the following formula [11]:

RC = 7 Vt + 5 (S + A) + 3 (Ds + Dr)(1)

where RC is the Forest Fire Susceptibility Index, Vt is vegetation type index, S is slope index, A is aspect index, Ds is distance from settlements index, and Dr is distance from roads index.

Each factor is analyzed separately and classified with the assigned values from 1 to 5, where 5 indicates the highest and value 1 indicates the lowest susceptibility to the occurrence of forest fires. Vegetation type is the most important factor for determining forest fire susceptibility. Coniferous vegetation, which contains resins and oils, is most susceptible to fire occurrence. Dry shrubs are also suitable for the occurrence of this disaster, while in broad-leaved forests, the probability is significantly reduced. Topography is an important physiographic factor that affects the fire proneness of the area [11]. Fire spreads faster at higher slopes by preheating, drying, and increasing the combustion of the available fuels in the canopy [32,33]. Fire travels most rapidly up steeper slopes and the least rapidly down slopes; therefore, terrain slope must be given significant importance in the determination of forest fire susceptibility. One aspect is another feature of topography that must be taken into account. It determines the amount of solar radiation that a particular area receives and affects the humidity of the vegetation.

Forests that are located next to roads, or have roads that pass through them, are more fire prone. Therefore, it is important to obtain data consisting of all roads in the research area. Here, not only paved or dirt backfilled roads used for vehicle movement are taken into account. The database should contain all the paths by which people can move through the forest, since human presence increases the probability of forest fire occurrence. Forests located near settlements are more vulnerable to fire, because people living there can cause an accidental fire [11]. All manmade structures, especially residential buildings, should be taken into account.

Since in Serbia, many forest fires each year occur near the agricultural areas, due to the widespread but very irresponsible custom of preparing agricultural areas for the new agricultural season by burning them, distance from such areas must be taken into account in order to perform adequate forest fire susceptibility zonation. Therefore, the existing Forest Fire Susceptibility Index formula must be modified, so that it contains the distance from agricultural areas index (Df). The lower-weighted value of the Df index stems from the fact that this is a protected natural area with a lower intensity of agricultural production. The new formula is:

RC = 7 Vt + 5 (S + A) + 3 (Ds + Dr) + 2 Df(2)

Table 1 shows the distribution of values on different classes of used parameters. Coniferous forest, terrain slopes with value over 35 degrees, slopes oriented to the south, and also areas that are the least remote to residential and other buildings types, roads, and agricultural areas are considered the most susceptible to forest fire occurrence.

Terrain slope and aspect were derived from a 25-meter cell size digital elevation model over Europe (EU-DEM) [34]. Slope, expressed in degrees, was then reclassified based on certain values with assignment of values from 1 to 5 to each class. Distance from residential buildings and other buildings types and roads was calculated according to data digitized from open satellite imagery. Vector data (buffer zones) were then rasterized to layer with the same cell size and projection of layers with data obtained from EU-DEM.

Vegetation type index was calculated on the basis of the random forest (RF) classification of Sentinel-2 multispectral imagery. Remote sensing technology can be used to obtain forest information from areas with rough terrain or that are difficult to reach. Therefore, it is necessary to explore the potential of remote sensing data to obtain adequate information about forest types [35]. The random forest (RF) algorithm is an influential method of collaborative learning developed for classification, regression, and unsupervised learning [36]. The objective of RF is to identify the appropriate model for analyzing the relationship between independent variables and a dependent variable for weight determination for each factor [1].

Sentinel-2 images Level-1C from 11 July 2017, 19 October 2017, and 22 April 2018 [37] were used for the purpose of obtaining the vegetation types in Nature Park Golija. With semi-automatic classification plugin (SACP) in QGIS software, the atmospheric correction of Sentinel-2 granules was performed, together with converting to reflectance, image mosaicking, and clipping to the nature park area. The following Sentinel-2 bands were used: blue (492 nm central wavelength), green (560 nm), red (665 nm), NIR (833 nm), and SWIR1 (1614 nm), for each aforementioned date. All bands have a spatial resolution of 10 m, except SWIR1 of which the spatial resolution is originally 20 m, but was automatically resampled to 10 m with SACP. Along with them, the Normalized Difference Vegetation Index (NDVI) and Bare Soil Index (BSI) were also used for each aforementioned date, in order to distinguish different types of vegetation more precisely, but also to distinguish artificial land cover and barren soil/rock. NDVI and BSI indexes are calculated as follows:

NDVI = (ρNIR − ρRed) / (ρNIR + ρRed)(3)

BSI = ((ρSWIR1 + ρRed) − (ρNIR + ρBlue)) / ((ρSWIR + ρRed) + (ρNIR + ρBlue))(4)

where ρBlue, ρRed, ρNIR, and ρSWIR1 are the reflectance from the blue band (B02), red band (B04), near-infrared band (B08), and shortwave-infrared band 1 (B11), respectively.

The training/test dataset consisted of 3.2 km^2^ digitized data classified in 8 classes: artificial areas, agricultural areas, grassland, shrub, broad-leaved forest, mixed forest, coniferous forest, and barren soil/rocks. Polygon data were transformed to point data, so they match 10 m pixels from the Sentinel-2 bands. Vegetation type data for Nature Park Golija were extracted based on RF in RStudio by the best combination of used Sentinel-2 bands, NDVI, and BSI, with an overall accuracy of 97.7% and a kappa coefficient of 0.97. Vegetation type index was obtained by the classification of random forest raster data according to Table 1. Distance from agricultural areas index (Df) was obtained from vegetation type data, by creating a vector layer with appropriate buffer zones of and rasterizing to layers with the same cell size and projection of layers with data obtained from EU-DEM.

### 2.2. Fuzzy AHP

Since the weight values for factors in the aforementioned forest fire susceptibility formula were pre-assigned, there is a justified need for a more objective distribution of the weighting values for each factor that is used for forest fire susceptibility determination. In order to obtain such distribution, the fuzzy AHP method was used. It should be emphasized that that in this case, same parameters with the same class values as for the Forest Fire Susceptibility Index calculation were used. The difference is in the weighted values of each factor used.

Fuzzy sets were introduced for the first time by Zadeh [38] in the form of object classes with a continuum of membership grades. Fuzzy AHP is a technique of incorporating vagueness or fuzziness of human thoughts in decision making [16]. In the fuzzy AHP model, a combination of AHP and fuzzy sets is used to weight the contributing factors in forest fire occurrence [10] and to model forest fire susceptibility. This modelling method uses expert ideas to express the importance and priority of each factor that contributes to forest fire occurrence. The fuzzy sets enter into the modelling process to express uncertainty and to obtain more accurate results than the ordinary AHP method [39].

The constituent of a fuzzy system is a fuzzy number. In this paper, for forest fire susceptibility assessment, a triangular fuzzy number was used, which is characterized by the form of the affiliation function μ(x). The triangular fuzzy number is defined by the values l_ij_, m_ij_, and u_ij_ (provided that l_ij_ ≤ m_ij_ ≤ u_ij_). The given values determine the boundaries of the fuzzy set, where the x-axis shows the size of the fuzzy number (left and right distribution), and the y-axis shows the degree of affiliation of the fuzzy number (between 0 and 1) [9,40]. The fuzzy AHP method involves the process of fuzzification of Saaty’s scale [41]. The conversion of Saaty’s numerical to the fuzzy AHP scale was performed using a triangular fuzzy number with a predefined confidence interval [40,42,43].

The first phase of fuzzy AHP method involves creating a fuzzy comparison matrix Ã at each level of the hierarchical structure using a triangular fuzzy number and fuzzy Saaty’s scale [10,44,45,46,47]. After creating the fuzzy comparison matrix, it is necessary to calculate the value of the fuzzy synthetic extent (Si) for each element [9,10,44,48,49,50]. In the next phase, calculation of the degree of possibility that M_2_ = (l_2_, m_2_, u_2_) ≥ M_1_ = (l_1_, m_1_, u_1_) should be carried out [9,45,49].

In the following, it is needed to analyze the degree of possibility that the convex fuzzy number is greater than the k convex number M_i_ (i = 1,2, …, k) and is defined by the expression [9,48,49,50].

V(M ≥ M_1_, M_2_, …, M_k_) = V[(M ≥ M_1_) and (M ≥ M_2_) and … and (M ≥ M_k_)] = = min V(M ≥ M_i_), i = 1,2, …, k (5)

Assume that

*d*′ (A_i_) = min V(S_i_ ≥ S_k_); k = 1,2, …, n; k ≠ i (6)

From the previous step arises the weight vector W’, and through normalization, the vector is reduced to the form W [9,48,49,50],

W′ = (d′ ^(A^_1_^)^, d′ ^(A^_2_^)^, …, d′ ^(A^_n_^)^)^T^ (7)

W = (d(A_1_), d(A_2_), …, d(A_n_))^T^ (8)

where W is a non-fuzzy number [9,48].

The final phase of the procedure represents the calculation of comparison matrix consistency. The comparison matrix, created using a triangular fuzzy number, needs a process of defuzzification. Further, it is needed to calculate the degree of consistency (CR), which represents the ratio of the index of consistency (CI) and the random index (RI), of which the value depends on the number of the compared criteria (n) [9]. The results of the comparison are considered consistent if the condition CR < 0.10 is met [9].

### 2.3. TOPSIS Method and Classification

In order to successfully analyze and compare forest fire susceptibility data obtained by applying these two methods, the TOPSIS method (technique for order of preference by similarity to ideal solution) is used. Its purpose is to optimize the process of ranking variant solutions from the most optimal to the least optimal, taking into account the characteristics of evaluation criteria and values of priority coefficients associated to them. It implies the existence of a decision matrix, and the initial step of the procedure involves the normalization of the decision matrix for each of the criteria, separately (r_ij_) [51,52,53]. The next step involves creating a weighted normalization decision matrix (a_ij_), where the values obtained in the previous step (r_ij_) and the values of the weight vectors (w_j_) are multiplied for each criteria [51,54].

(9) $$r_{\mathrm{ij}}=\frac{x_{\mathrm{ij}}}{\sqrt{\sum_{j=1}^{n}x_{\mathrm{ij}}{}^{2}}}$$

(10) $$a_{\mathrm{ij}}={\;w}_{\mathrm{ij}}\frac{x_{\mathrm{ij}}}{\sqrt{\sum_{j=1}^{n}x_{\mathrm{ij}}{}^{2}}}$$

Third step involves the identification of ideal variant (V_j_+) and anti-ideal variant (V_j_−) values for each criterion. These values are directly related to the goal of the analytical procedure and the nature of the criteria, i.e., they depend on whether maximization or minimization is performed for a certain criterion during the evaluation [52,55].

(11) $$V_{j}+=\left[\left(\max V_{\mathrm{ij}}\;|\;j\;\in \;J\right),\left(\min V_{\mathrm{ij}}\;|\;j\;\in \;J\prime \right)\right]$$

(12) $$V_{j}-=\left[\left(\min V_{\mathrm{ij}}\;|\;j\;\in \;J\right),\left(\max V_{\mathrm{ij}}\;|\;j\;\in \;J\prime \right)\right]$$

Identification of the ideal and anti-ideal ranked variants is performed in order to calculate the value of the geometric distance of each of the variant solutions from the mentioned values, i.e., in order to identify the values of deviations represented by *d_i_*+ *i d_i_*− indicators [51,52,53,55,56].

(13) $$d_{i}+=\sqrt{\overset{n}{\underset{j=1}{\sum }}{\left(V_{\mathrm{ij}}-V_{j}+\right)}^{2}}$$

(14) $$d_{i}-=\sqrt{\overset{n}{\underset{j=1}{\sum }}{\left(V_{\mathrm{ij}}-V_{j}-\right)}^{2}}$$

In the final phase, the value of the performance index (cl_i_) is calculated for each of the variant solutions, in order to rank them from the most optimal to the least optimal [51,52,54,55,56].

(15) $${\mathrm{cl}}_{i}=\frac{d_{i}-}{\left[\left(d_{i}+\right)+\left(d_{i}-\right)\right]}$$

Values of the performance index are in range from 0, for the most susceptible to forest fire occurrence, to 1, for the least susceptible. Zones of different forest fire susceptibility for the research area were classified based on the value of performance index according to Table 2.

### 2.4. Adopted Fuzzy AHP Methods for the Forest Fire Risk Assessment and Outbreak Detection

**Figure 2:**
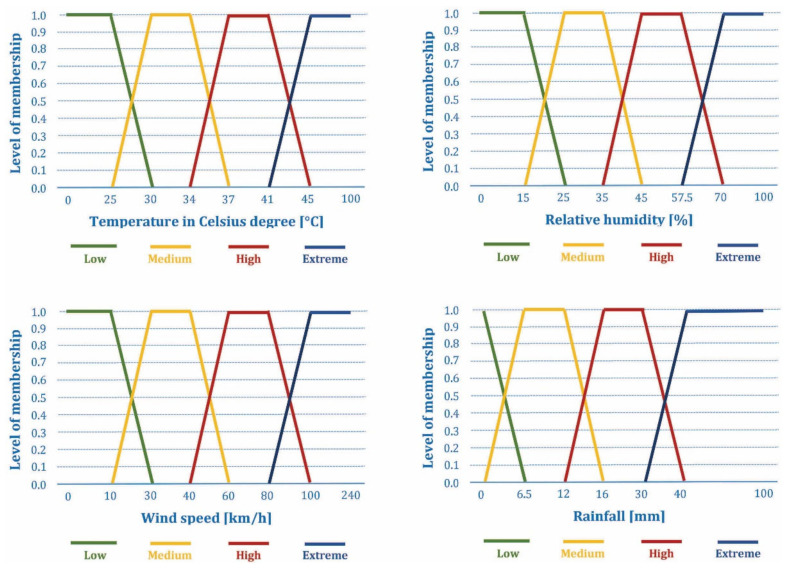
Membership functions of meteorological variables, adopted from [57].

**Figure 3:**
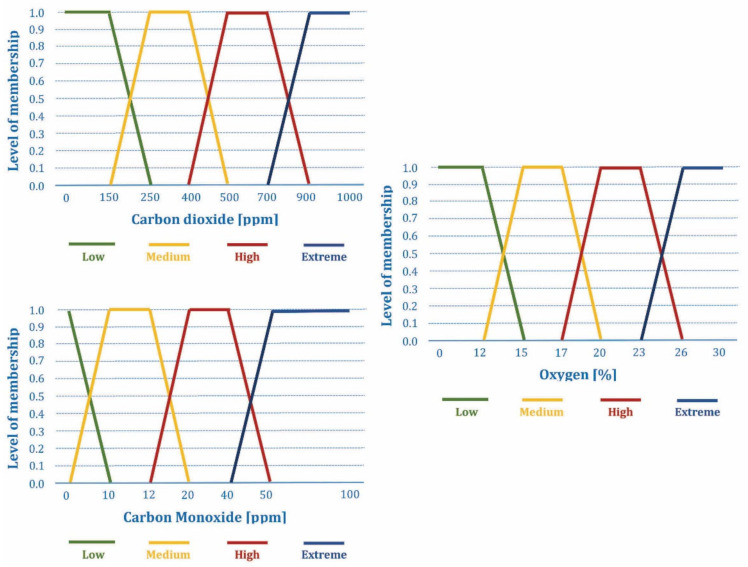
Membership functions of chemical gases, adopted from [57].

The adopted fuzzy AHP method, as discussed in Section 4.1, is based on the measuring the meteorological parameters (temperature, relative humidity, wind speed, and rainfall) and the concentrations of the chemical gases (oxygen, carbon monoxide, and carbon dioxide) that are responsible for forest fire ignition. This real-time environmental monitoring of the aforementioned forest fire risk factors is performed by a distributed and IoT-based sensor network. Corresponding sensors are deployed in the given area, with the decreasing node density as the forest fire susceptibility decreases, considering the results of analysis with the fuzzy AHP method presented in Section 3. The sensor node location is considered known due to planned deployment (i.e., the information could be written in sensor node software, or the unique sensor node ID could be defined with the corresponding location information written in the system database). Alternatively, each sensor node can be equipped with a Global Positioning System (GPS) module, which would increase the power consumption and cost of the sensor node. Each sensor periodically collects the measured data and employs the fuzzy logic Mamdani inference system [57,58] that has been adopted to analyze the complex environmental changes. Seven input variables measured by sensors are fuzzified using the four membership functions shown in Figure 2 and Figure 3. Different fuzzy sets are defined for each linguistic variable expressed as “very low”, “low”, “normal”, “high”, and “extreme”, which is presented in Table 3.

The adopted fuzzy sets for the forest fire risk are designed according to the rule of 30, which is considered as a relevant preventive model of forest fire risk [57]. This rule considers measurements of temperature and wind speed above 30 °C and 30 km/h, respectively, with the humidity values below 30%, as the environmental conditions that favor the forest fires’ occurrence. The adopted fuzzy sets for the fire outbreak occurrence are proposed and designed, [57], based on the occurrence of the unusual increases in carbon monoxide and carbon dioxide levels above their typical environmental concentrations at forest areas, at the same time as the occurrence of the unexpected oxygen level decrease below 21%.

Every measured environmental variable is compared with its average value. The number of consecutive measurements used to calculate this average value depends on the current environmental conditions as summarized in Table 4. A comparison between the last environmental measurement and its average is necessary to evaluate unusual environmental changes and predict fire occurrence.

The proposed fuzzy inference system has two output variables, i.e., forest fire risk estimation and forest fire outbreak detection for the fire prevention and detection purpose, respectively. In order to prevent fire occurrence, fuzzified values of the last measurement and its average for meteorological variables (temperature, relative humidity, wind speed, and rainfall) are compared with the aim of evaluating the existence and severity of forest fire risks in the particular zone. Therefore, the considered output linguistic variable is the existence of forest fire risks expressed as “nonexistent”, “low”, “high”, and “extreme”. In order to detect fire occurrence, fuzzified concentrations of chemical gases (oxygen, carbon monoxide, and carbon dioxide) are compared with the respective average values in order to evaluate the probability that the fire outbreak recently occurred. The same output linguistic variables were used to calculate the probability of fire detecting as for fire prevention.

**Figure 4:**
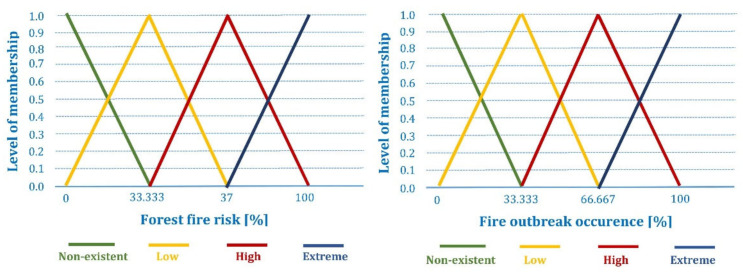
Membership functions of output variables, adopted from [57].

After application of the corresponding inference rules, the results obtained for the input linguistic variables are aggregated into two output fuzzy sets and fuzzified into the output membership functions showed in Figure 4.

Both output fuzzy sets are defuzzified with the centroid method [59], which calculates the center of mass for the obtained output distributions. Hence, a non-fuzzy discrete percentage of the forest fire risks and fire outbreak occurrence represents the result for the fire prevention and detection tasks, respectively.

## 3. Results

### Forest Fire Susceptibility Zonation

**Figure 5:**
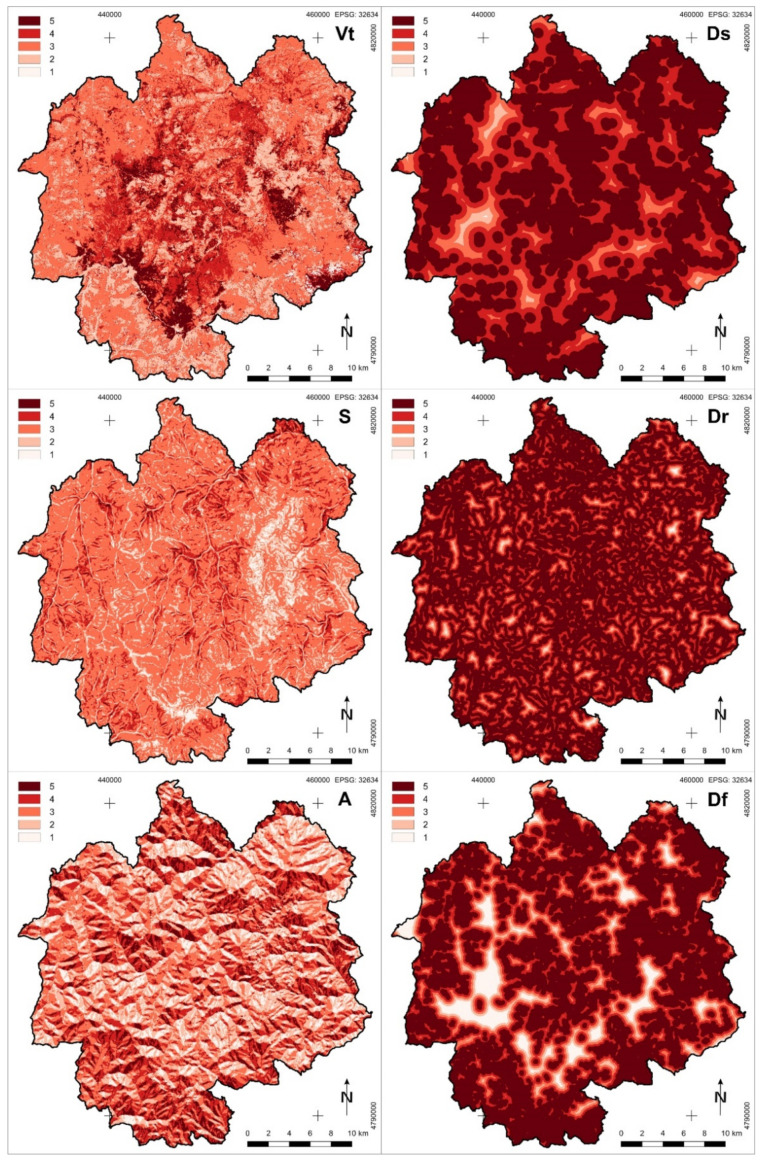
Spatial distribution of classes in factors in Nature Park Golija.

By analyzing data from layers that were used for forest fire susceptibility calculation, it has been determined that coniferous forests, that are the most susceptible to forest fire occurrence, cover 9.4% of total Nature Park Golija area, and mixed forest and shrub, that is also highly susceptible, covers 22.3% of total area. The largest part of the nature park is covered by broad-leaved forest (48.5%), which is moderately susceptible to forest fire occurrence (Table 5, Figure 5).

Slopes steeper than 35°, which are the most prone to fires, occupy 1.3% of the total research area. Highly prone areas with terrain slope between 25° and 35° are found on 13.2% of the nature park, and moderately prone terrain with steepness from 10° to 25° covers the remaining 66.8%. South-oriented hillsides cover 10.3% of the total area. The share of other classes of slope, aspect, as well as distance from buildings, roads, and agricultural areas, in the total nature park area can be found in Table 5, and their spatial distribution is shown in Figure 5.

For the calculation of the Forest Fire Susceptibility Index, predefined weighted values of factors were used. For the calculation of forest fire susceptibility using the fuzzy AHP method, a fuzzy comparison matrix was created (Table 6). For such comparison matrix, consistency ratio (CR) equals 0.055; therefore, the results of the comparison are considered as consistent. After the calculations weighted values (w_j_) of factors were obtained, which are presented in Table 7.

**Figure 6:**
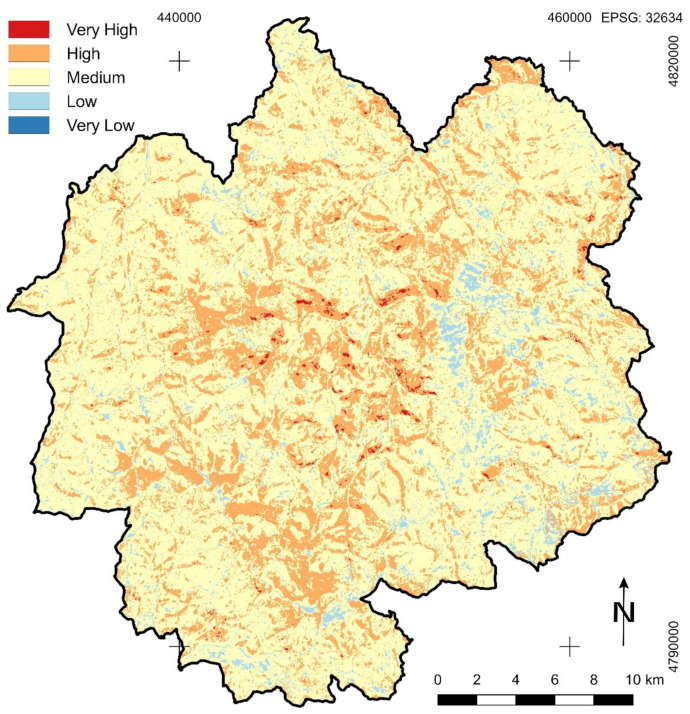
Forest fire susceptibility zones in the Nature Park Golija, according to the Forest Fire Susceptibility Index method.

According to results obtained by using the index-based RC method for forest fire susceptibility zonation very high susceptibility is represented on 0.5% of the total research area. High susceptibility was determined on 24.2% of the territory. Medium susceptibility is represented on 70.5% of the nature park area, and low on 4.8%. Area on which very low fire susceptibility is determined is almost unnoticeable, which can be seen numerically in Table 8 and graphically in Figure 6.

**Figure 7:**
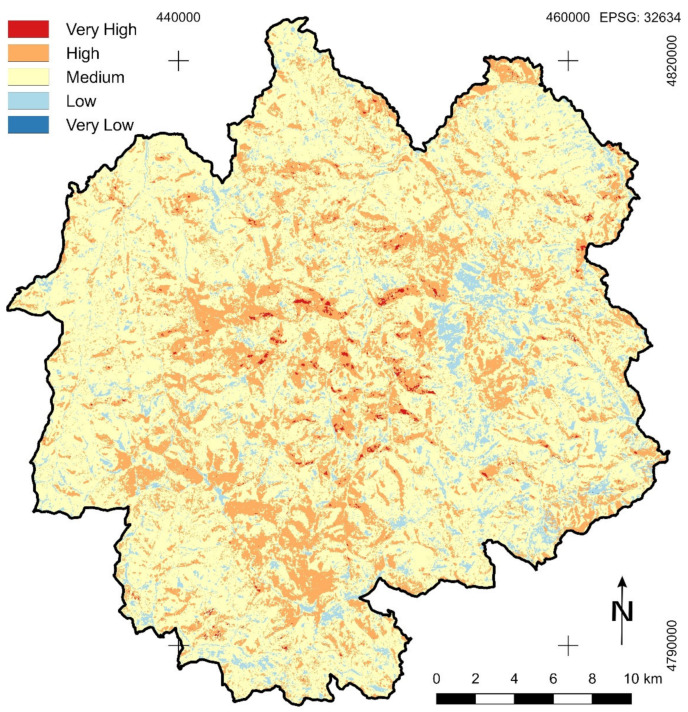
Forest fire susceptibility zones in the Nature Park Golija, according to the fuzzy AHP method.

Because of similar mutual relation of weight coefficients that were calculated by using the fuzzy AHP method and using the TOPSIS method for normalization and classification of the results of both methods to determine forest fire susceptibility of the study area, the results obtained by using the fuzzy AHP method are not much different from the results obtained by using the previous method (Table 8, Figure 7). It was noted that there was a slight increase in the area of very high susceptibility and also areas with very low susceptibility. The biggest absolute difference was in the area covered by the zone of low susceptibility. It can be said with great certainty that the results obtained by usage of the fuzzy AHP method are more accurate. Areas with very high forest fire susceptibility are the most present in the central parts of NP Golija, which are areas with coniferous or mixed forest vegetation, south oriented steeper slopes, and with a significant share of anthropogenic factor.

When forest fire susceptibility zonation is analyzed from the protection zone point of view in Nature Park Golija, it is determined that in areas in the first regime of protection, which is most important for protection of nature and thus most vulnerable, 0.1% of total areas in that zone are very highly susceptible to forest fire occurrence, and 32.0 to 32.4% of areas are highly susceptible, depending on the applied method. In the second zone of the protection regime, 0.7% of total areas are classified in the class of very high susceptibility and about 34% in class of high susceptibility (Table 9). What is encouraging is that the share of the zone with very high susceptibility to forest fires in the areas with the first protection regime is significantly lower than in other regimes. On the other hand, the share of the high susceptibility zone is significantly higher in the first protection regime than in the entire nature park on average, which is why much more attention must be paid to fire protection in such areas. For the areas in the second regime of protection, both very high and high forest fire susceptibility classes are present in larger percentages than in the total nature park area. This means that areas important for nature conservation are much more vulnerable taking everything that is significant for forest fire risk management into account.

By analyzing forest fire susceptibility in the area that belongs to MaB Reserve Golija-Studenica, it has been determined that about 0.6% of the total reserve area belongs to the class of very high susceptibility, 25.2 to 26.3% to the class of high susceptibility, depending on the applied method, and 68 to 69.2% to the medium forest fire susceptibility class (Table 10). The higher share of the zone with very high susceptibility indicates the greater importance of the protection of the reserve area.

## 4. General Description of Adopted IoT-Based System for the Forest Fire Monitoring

In this section, we give a general description and rationale for the adopted IoT-based system, which enables the continuous collection of the meteorological data for the purpose of improved forest fire susceptibility assessment in a long-term and short-term manner and the real-time prevention and detection of forest fire in the Nature Park Golija. This system must support all aforementioned functionalities, but also operate in the harsh environment of Nature Park Golija under the conditions of the lowest possible influence on the natural environment and wildlife in the given area. Additionally, the previously presented results of forest fire susceptibility analysis by using the fuzzy AHP method (in Section 3) are taken into the account regarding system architecture and the sensor node deployment strategy.

In order to support intended functions, the proposed system must contain the large number of sensor nodes deployed in the given area. Due to the non-existent power grid in the area and the difficult maintenance conditions (i.e., battery change), the sensor nodes must operate on battery power supply, while these energy-constrained nodes could eventually be equipped with solar panels in order to prolong operating life at the cost of a higher sensor node unit and network price, [60]. Additionally, due to the lack of communication infrastructure, a natural choice to achieve sensor nodes’ connectivity in the given large-scale sensor network and under the given environmental circumstances is the adoption of wireless communication technologies that can support long-range communication and low energy consumption. This leads to the choice of low-power wireless communication technologies characterized with the energy-aware protocols and sets the demand to adopt the lowest possible data rate between sensor nodes and central elements of the system that are needed to support the given application demands. Therefore, the obvious choice is to adopt the system architecture and operating principles in which all tasks that can be achieved by the local processing in the sensor nodes, without the frequent communication of measured data to the other network elements, should be realized in the edge processing manner (i.e., in sensor nodes). In this case, sensor nodes will only communicate the final results to the central elements of the system, thus minimizing the communication demand and data rate in the network and overall energy consumption. However, this should be done in a manner that provides all necessary environmental sensory data needed in the central element of the system (i.e., in the cloud).

In the following sub-section, the several design choices are presented with the appropriate discussion and explanation.

### 4.1. The Choice of Environmental Parameters for the Forest Fire Risk Prediction and Detection

In the case of the real-time early forest fire prediction and detection system, the main demand is that it should be able to notify firefighters as quickly as possible in order to minimize damage to wildlife, environment, infrastructure, and people caused by the fire. In that sense, the most important requirements of the forest fire detection systems include [61]: robust continuous monitoring, fast detection of fire outbreak, determination of the exact location of fire, fast notification, and minimization of probability of false alarms occurrence. The comprehensive state-of-the-art analysis of previously proposed forest fire prediction and detection solutions shows that this system can be classified in several categories [61,62]:

- Traditional human-based observation systems;
- Satellite-based systems;
- Optical and thermal sensors (cameras)-based systems;
- IoT-based sensor networks and wireless sensor networks (WSN)-based systems;
- Unmanned aerial vehicles (UAV)-based systems.

As defined in the Introduction, the main task of the sensor network observed in this paper is the continuous measurement of meteorological parameters that can be collected and used for the forest fire susceptibility assessment, while the real-time forest fire prediction and detection is considered as a secondary (additional) task. In that sense, the main meteorological parameters that should be gathered and delivered for the forest fire susceptibility assessment are [17,18,19,20]: temperature, relative humidity, wind speed, and rainfall.

Of all aforementioned systems, only the WSN and IoT-based sensor networks-based systems inherently contain sensor nodes equipped with the set of sensors that measure temperature, relative humidity, wind speed, and rainfall. The operating principles of the other systems demand the use of other sensor types, i.e., video cameras sensitive to the visible spectrum of smoke during the day and a fire during the night, infrared (IR) thermal imaging cameras, IR spectrometers able to identify smoke, or light detection and ranging systems (LIDAR) in optical and thermal sensor-based systems and UAV-based systems. Thus, all systems, except WSN- and IoT-based sensor networks-based systems are considered to be out of scope of this paper, although some of them may have higher performance for the forest fire detection purposes under the given circumstances. Of course, here, the adopted system can be complemented with different forest fire detection components, but it would cause additional costs.

The literature review of IoT-based sensor networks and WSN-based systems intended for the real-time forest fire risk prediction and fire outbreak detection showed the broad set of possible meteorological and other parameters used in these systems. The following is observed:

- The most commonly used set of parameters consists of: temperature (T), relative humidity (H), wind speed (W_speed_), and rainfall (R), [17,18,57,63,64,65,66,67,68,69,70,71,72]. For this set of parameters, the fuzzy AHP method is used [57,63,64,69] for the forest fire risk prediction, sometimes with additional parameters for human behavior and environment [64], while these same parameters were also combined with the concentrations of chemical gases (oxygen, carbon monoxide, and carbon dioxide) in order to detect forest fire outbreaks [57]. Additionally, the same set of parameters is used as an input for artificial neural networks (ANN) [66,68] or linear regression model [70], or to calculate the fire weather index (FWI) [65,67,71,72] by using a pre-defined procedure, for the purpose of forest fire risk prediction.
- The narrowed set of parameters that include temperature and relative humidity is proposed for the forest fire risk prediction in combination with other sensors, such as vision sensors [73,74] with fusion realized using Dempster–Shafer evidential theory, smoke and light intensity sensors [75,76], or light intensity and carbon monoxide sensors [77] by using the fuzzy AHP method. The combined measurements of temperature, relative humidity, and carbon monoxide are employed for forest fire risk prediction with broad set of machine-learning algorithms [78], while the combined measurements of temperature, relative humidity, carbon monoxide, carbon dioxide, and smoke sensors are used as input in ANN [79,80].
- A several simple solutions were proposed in which temperature measurements are combined with the measurements of smoke sensors or combustion sensors (i.e., combined carbon dioxide and nitric oxide measurements) [81,82], or only temperature measurements are used [83], for the forest fire detection by using ANN. Additionally, a combination of temperature and carbon monoxide measurements is used in a simple forest fire detection system [84] with the predefined decision criteria.
- Finally, a very complex solution in which soil parameters (soil temperature, soil moisture), air parameters (air temperature, air moisture, carbon dioxide concentration, wind direction, wind speed, precipitation, negative oxygen ion, PM 2.5), light parameters (global radiation, light intensity, sunshine hours threshold, photo synthetically active radiation), and plant parameters (needle-type stem moisture, non-destructive stem moisture) is used for forest fire prediction [85].

Considering that the basic meteorological parameters (T, H, W_speed_, and R) must be collected, and as a primary task of sensor network, we adopted the following set of meteorological parameters (temperature, relative humidity, wind speed, rainfall) for the real-time forest fire risk prediction, and in order to improve the performance of the real-time fire outbreak detection, a set of sensors for chemical gases concentration (oxygen, carbon monoxide, and carbon dioxide) is added. Additionally, by analyzing the presented wide range of proposed solutions for the forest fire prediction and detection, as well as the fact that there are no corresponding datasets available for the given area (that contain real-time measurements labeled with the information on the fire outbreak occurrence), which are needed for the training of ANN models or machine-learning algorithms, we here adopted the fuzzy AHP method. Therefore, the final proposal similar to the one presented in [57] is adopted for the real-time forest fire risk prediction and fire outbreak detection, which includes a set of aforementioned seven parameters, with the fuzzy AHP method being employed for the both tasks. More details are given in Section 2.4.

Finally, the same set of meteorological parameters (temperature, relative humidity, wind speed, rainfall) is considered for the purpose of long-term and short-term forest fire susceptibility assessment.

### 4.2. The Choice of Communication Technology for the IoT-Based Sensor Networks for Forest Fire Prediction and Detection

The application of wireless sensor networks (WSN) that encompass a large number of Internet of Things (IoT) devices [86] in real-time forest fire prediction and detection has been proposed more than 15 years ago [87], with the argument that WSN can forecast forest fire in a more efficient manner than the traditional satellite-based approaches [88]. However, WSN deployment in a large-scale forest area, such as NP Golija, imposes certain challenges related to the power supply, maintenance, coverage, and infrastructure cost. Short-range wireless technologies such as Wi-Fi, Bluetooth, and ZigBee [60,89] can use less complex sensor nodes for fire detection systems but close to the inhabited areas. On the other hand, long-range 3GPP technologies such as GSM, UMTS, and LTE can provide Narrowband Internet of Things (NB-IoT) functionality [90] with the extended coverage, but also require higher end-device complexity, which consumes more power and license for frequency spectrum utilization. It is worth mentioning that substantial financial resources are needed for the additional optical infrastructure, microwave links, or base stations if the forest area of interest lacks cellular or Wi-Fi coverage. The analysis of cellular networks coverage in the area of interest, in NP Golija, shows that the existing networks do not offer complete coverage. Due to the low economic interest of cellular network operators and the strict environmental conditions that must be met in the protected areas of NP Golija, the setting additional base stations in the area present an issue. A hybrid solution comprised large-scale WSN connected to a cellular network via a corresponding gateway seems to be a good trade-off between the aforementioned conflicting requirements. Nevertheless, this network setup still requires dense WSN deployment with limited inter-sensor distance and satisfactory cellular radio conditions at gateway locations.

Long-range wide-area network (LoRaWAN) technology [60,91,92,93] presents a good candidate for this kind of application, since it supports both large coverage, which decreases the number of required sensor nodes, and low power consumption, which prolongs their lifetime. Moreover, LoRaWAN standard utilizes unlicensed industrial, scientific, and medical (ISM) frequency low band with favorable radio conditions [94] and is based on open protocols [93]. Hence, the application of LoRaWAN allows for customization and large-scale deployment without dependence on regulator or standardization body. The considered application demands low data rate, low power consumption of sensor nodes, and long-range communication. Under these conditions, LoRaWAN technology seems to be the most appropriate solution for wireless connectivity of sensor nodes [60,92,93], in comparison to short-range and other long-range alternatives. Additionally, in LoRaWAN networks, each sensor can be served by multiple gateways in the area. With LoRaWAN, each uplink packet sent by the end-device will be received by all gateways within reach. This arrangement significantly reduces the packet error rate (since the chances that at least one gateway will receive the message are very high) and allows for low-cost geolocation. The LoRaWAN technology also supports high security, which is of great importance for the considered application.

The concept of the forest fire detection using LoRaWAN sensor networks is introduced in [95] and subsequently experimented in laboratory conditions [81] and verified in a real network deployment scenario [60,96]. These LoRaWAN forest fire monitoring systems gather environmental data collected from LoRa sensor nodes via the LoRa gateway to the corresponding application server where the main data processing is carried out. The fire estimation procedure is based on the 30–30–30 rule, which is not the most efficient method, since a forest fire sometimes depends on the area where it is placed and its topography [97]. Fuzzy logic and its analytic hierarchy process (AHP) method [98] can be used here to enhance real-time environmental data analysis. Fuzzy theory is applied when analyzing the causation of uncertain factors, which corresponds to this specific use case. Seminal works [70] in this area developed fuzzy logic algorithms that estimate the probability and direction of fire based on the status of membership functions that utilize measured sensor data. Combined analysis of measured and historical data can significantly increase forest fire prediction and detection accuracy. Further research [57,63,64] considered human behavioral (date and time) and environmental (road and population density) factors together with weather data obtained from sensors as an input for their fuzzy reasoning system, which gives the probability of fire occurrence. The authors in [57] deployed an AHP-based fire spread estimator, which combines measured data with additional static forest data such as water resources, landscape position, forest tracks, and vegetation to better estimate the forest fire propagation once a fire outbreak is detected.

Considering the previous discussion, in this section, we adopted the IoT-based sensor network for forest fire prevention, prediction, and detection, with the access network realized in the form of LoRaWAN system architecture. As already stated, the fuzzy AHP method is applied for the real-time tasks (forest fire risk prediction and fire outbreak detection) and short-term and long-term forest fire susceptibility assessment, which utilizes both real-time (i.e., collected by sensor networks in real-time) and static data.

### 4.3. Overall System Description

Considering the previous discussion, we here propose the IoT-based system for the real-time prevention, detection, and tracking of forest fire in the area of interest, which also supports the continuous collection of all necessary meteorological parameters (temperature, relative humidity, wind speed, rainfall) needed for the forest fire susceptibility assessment in a long-term and short-term manner (by using existing data already used for the forest fire susceptibility assessment described in Section 2 and the result shown in Section 3).

The first part of the system in an IoT-based sensor network consists of a large number of sensor nodes that measure the local meteorological parameters (temperature, relative humidity, wind speed, rainfall) and the concentration of chemical gases (oxygen, carbon monoxide, and carbon dioxide) that are responsible for forest fire ignition. These sensor nodes are equipped with sensors and are dispersed in the area of interest. Sensor nodes should be deployed with the decreasing node density as the forest fire susceptibility decreases, considering the results of analysis with fuzzy AHP method presented in Section 3. As evident from this analysis, less than 0.5% of the total NP Golija area belongs to the very high forest fire susceptibility class, while around 24% and 70% of the total NP Golija area belong to the high and medium forest fire susceptibility classes, respectfully. The low and very low forest fire susceptibility classes cover much less percentage of total NP Golija area. In order to ensure proper operational conditions for the proposed system, the relative number of sensor nodes deployed in the same surface area must be dependent on the forest fire susceptibility classes, in the ratio 5:3:2:1:1 for the very high, high, medium, low, and very low susceptibility classes, respectfully. The planned deployment of a sensor node enables a priori knowledge of their location, even without a GPS module. As discussed before, each sensor node can be additionally equipped with a GPS module, which increase flexibility in sensor node deployment and operation, but at the cost of the increased power consumption and network cost. The location of sensor nodes should be carefully chosen in the areas in which there are the most favorable conditions for the fire outbreak (i.e., in the areas covered with pines and other coniferous trees, near the roads and picnic areas, on the slopes facing south, etc.).

The sensor nodes are equipped with LoRa radio interface modules in order to establish bidirectional communication with the LoRaWAN gateways deployed in the area. The required number of LoRaWAN gateways must be defined through radio planning process so that the proper radio and service coverage is achieved (the coverage of gateways is partially overlapped in order to enable multiple reception of sensor nodes messages for the border regions in order).

Each sensor node periodically performs measurements and employs the fuzzy AHP method described in Section 2.4 adopted to analyze the complex environmental changes in order to calculate two output variables, i.e., forest fire risk estimation and fire outbreak detection, for the real-time fire prevention and detection purpose, respectively. If the percentage of the estimated forest fire risk is high, the node will send this information with its location or ID towards the central element of the system (i.e., to the cloud server), which will log this event and notify the emergency team through its corresponding web platform. If the percentage of fire outbreak occurrence is also high, the node will send the same information to the server but this time with the additional fuzzified input of all meteorological variables in order to enable creation of appropriate database inputs of such occurrences for the sake of further improvements of the system. Additionally, an optional real-time task might be realized based on the aforementioned data. That is, further processing might be performed by the central server by using the corresponding data together with already stored information about vegetation type, slope index, and sensor location, in order to calculate wind direction and predict the fire spread direction by using the method defined in [57]. In both scenarios, values of the meteorological variables and chemical gases concentrations related with fire prevention or detection procedures will be stored in the central database for future purposes.

One possible variant in the areas with the higher sensor node density is to organize sensor node in clusters so that each sensor node has a corresponding cluster head, i.e., sensor node equipped with a LoRa interface (LoRa node), which is deployed in the particular zone. The cluster members perform the same tasks as in the previous case (when all nodes are LoRa nodes) and send their messages through the wireless communication with the cluster head (i.e., LoRa node) achieved by using short-range communication technologies, e.g., ZigBee technology. The LoRa node in this case performs data aggregation and delivers all data for its cluster to the central server.

Each sensor node sends a daily report with all the sensory data in standard format for the meteorological parameters (a similar format can be used for data on gases), in order to update the corresponding long-term database on a daily basis. These data are used for the long-term and short-term forest fire susceptibility assessment. In case of the long-term assessment (i.e., monthly or yearly), collected meteorological parameter data (historical data) are averaged and included in the fuzzy AHP method described in Section 2.1, Section 2.2 and Section 2.3 in its final sub-layer, similarly as in [17,18,19,20]. Thus, the overall long-term forest fire susceptibility assessment will take into account local meteorological conditions. This will also ensure proper assessment and fire prevention activities in the long term, based on the local and more appropriate meteorological data. For the improved short-term (daily) forest fire susceptibility assessment, we here propose that the daily reported meteorological data are used to calculate the fire weather index (FWI) solely by four meteorological variables as input ones (i.e., temperature, wind speed, relative humidity at noon time, and accumulated precipitation during previous 24 h period) [17,65,67,71,99]. The FWI system produces six indices based on the referenced fuel type, that is: (1) fine fuel moisture code (FFMC) that is calculated by using a function of temperature, wind speed, relative humidity, and precipitation; (2) duff moisture code (DMC) that is calculated by using a function of temperature, relative humidity, and precipitation; (3) drought code (DC) that is calculated as a function of temperature and precipitation; (4) initial spread index (ISI) that is calculated as a function of FFMC and wind speed; (5) buildup index (BUI) that is calculated as a function of the DMC and DC; (6) FWI that is calculated as a function of ISI and BUI. Alternatively, FWI can be calculated by using the fuzzy inference method defined in [69]. So, calculated FWI values can be included in the fuzzy AHP method described in Section 2.1, Section 2.2 and Section 2.3 in its final sub-layer, i.e., similarly as in [17], in order to achieve short-term forest fire susceptibility assessment. For the long-term and short-term forest fire susceptibility assessment, the calculation for each elementary area is performed by using meteorological data averaged over the closest sensor nodes.

The long-term deployment of a proposed IoT-based system would enable the collection of historical, meteorological, and other data. Such a database enables further research, especially if all fire occurrence in the area is properly documented. This will enable the application of more sophisticated inference methods (i.e., machine learning or deep learning) that could further improve the forest fire prevention performance of the overall system.

## 5. Discussion

The International Strategy for Disaster Reduction [100] encourages researchers to promote the protection of the environment in order to reduce vulnerability to disasters. Previous research has shown that protected areas can play a very important role in preventing or mitigating natural disasters [101,102]. Assessment and zoning of natural disaster susceptibility, vulnerability, hazard, and risk are among the most important parts of natural disaster management and mitigation. Therefore, it is necessary to constantly improve necessary methods and to find new data sources that could be used for that purpose. Climate change trends are such that the forest fire risk is likely to increase globally in the near future. Most of the world countries will be affected that way. Many of them that are not considered as wealthy and will have problems in dealing with this disaster because of lack of funding for data and equipment, among other things. That is why it is important to consider different possibilities, methods, and datasets, which will be more affordable to most parts of the world. The consequences of forest fires in protected natural areas, due to their importance and characteristics, are the reason why forest fire management and mitigation must be at the top of the priority list.

The application of GIS is indispensable for this type of research, because only with its help can an adequate and complete spatial analysis be performed. All the necessary parameters that are important for forest fire susceptibility assessment can be taken into account. Together with remote sensing, GIS represents a very powerful tool that enables the use of various methods and multi-criteria decision analysis.

The data and software that were used in this paper for forest fire susceptibility assessment and zonation are completely open source. That kind of research could help communities that have a low budget when it comes to nature protection and forest fire risk management. This paper also shows that it is possible to perform suitable forest fire susceptibility zonation even if historical data of forest fires that occurred in a certain area and relevant meteorological data are not available. Of course, for comprehensive forest fire risk assessment, more accurate datasets than those used for this research and also historical forest fire occurrence, climate, and weather data should be considered. Not only that it would provide more detailed results of forest fire risk assessment and zonation, even before setting up a sensor network, but it would also allow more advanced methods to be used, such as the application of artificial intelligence.

As already stated in the Introduction, the most significant contribution of this research is that the fuzzy AHP method was used for the first time for forest fire susceptibility assessment in Serbia. The fact that it was used on the territory of one of the most important protected natural areas amplifies that significance. Additionally, it compares results of the index-based method and fuzzy AHP using the TOPSIS method. The application of the fuzzy AHP method is more adequate, because it is more objective. Although at first glance there were no significant differences in the results of the applied methods, these differences are still significant for the further design of the sensor network based on the obtained results, as well as for the overall management of this disaster. For future research and improvement of methods and results, using other techniques that give adequate results, such as the fuzzy analytical network process (ANP) [15], or hybrid approaches [103], should be considered.

The results obtained thanks to such research should be an integral part of spatial plans and other planning documents. They should help those who manage protected natural assets, such as the Nature Park Golija, to reduce the forest fire risk, as well as to prevent possible consequences. The significance of this research is that it is not only applicable to protected natural assets, but can also be applied to other spatial units. The research should benefit state and local authorities, emergency management services, but also the local population.

Another important goal of research presented in this paper, as stated in the Introduction, was to propose adequate system architecture and an underlying sensor network in order to enable the continuous collection of local meteorological data in order to amend and improve the forest fire susceptibility assessment performance achieved with the aforementioned fuzzy AHP method (which does not use meteorological data). The main design requirements for such a system were to enable gathering of local meteorological data, i.e., data collected by the sensors densely deployed in area of interest, in order to further improve the quality of the forest fire susceptibility assessment in a long-term manner (i.e., monthly or yearly by using averaged data) or in a short-term manner (i.e., daily by using current data). Additional goals included system ability to perform real-time forest fire risk assessment and fire outbreak detection, and to gather all necessary data for the future research and improvements of the overall forest monitoring system, and the future use of more advanced inference methods such as machine-learning and deep-learning models. The specific characteristics of the chosen area (NP Golija) set additional demands regarding power supply and efficiency, maintenance, use of long-range low-data-rate wireless technology. Additionally, the proposed system had to be cost effective (as it was intended for the large-scale usage and should be appropriate for the countries with the lower possible budget for these purposes). The above requirements, as elaborated in Section 3, set a specific set of demands in the sensor network and the overall system design.

Therefore, the comprehensive state-of-the-art analysis of possible system architecture and sensor network operating and design principles was conducted, with the main results of that analysis presented in Section 3 through the elaborated discussion regarding the choice of environmental parameters, which should be monitored, and the choice of communication technology and architecture that is most suitable for the given application requirements. The main choice was to include a set of weather and environmental parameters (temperature, humidity, rainfall, wind speed, as well as carbon monoxide, carbon dioxide, and oxygen levels) for the long-term and short-term forest fire risk assessment (as an additional part of the fuzzy AHP method based on the other variables) and the real-time forest fire risk estimation and fire outbreak occurrence detection. We proposed modern and advanced system architecture, in the form of an IoT-based sensor network with LoRaWAN technology used in the access part (sensor network part) of the system in order to achieve reliable, low-rate, low-power, low-cost, and long-range bidirectional communication of sensor nodes. The supported security, reliability, and traffic demands in this network absolutely satisfies all current and future demands and also enables the design of low-cost and long-life sensor nodes for a large-scale, cost-effective, and low-maintenance system. It can be argued that there are other possible solutions for the real-time forest fire risk prediction and outbreak detection, such as those based on optical and thermal sensors or UAV. This kind of system might offer better real-time forest monitoring performance than the system adopted in this study. However, these systems do not include the ability for the long-term collection of meteorological and environmental data, which was one of the tasks, and can be viewed only as a possible additional segment, which would significantly increase system cost and complexity. Thus, the proposed system architecture and sensor network part fulfill all application requirements with the lower overall cost.

Finally, it should be noticed that the proposed general framework for an IoT-based sensor network and the overall system presents only one possible solution. However, based on the comprehensive analysis of so-far-proposed solutions, as was elaborated in the previous sections, we concluded that this solution represents the most suitable framework for design and deployment under the given requirements, especially the demand for a low-cost solution. Of course, under different circumstances, i.e., larger possible budget, other more advanced solutions could be envisioned.

## 6. Conclusions

Forest fire susceptibility zoning is one of the first and one of the unavoidable steps in forest fire risk reduction and mitigation. It makes it possible to obtain data on where forest fires are more likely to occur and to identify certain “hot spots” where their occurrence is most expected. Based on that, decisions can be made that will prevent possible consequences, save the lives of people and other living beings, and preserve tangible and intangible goods. Forest fire mitigation is especially important when it comes to protected natural areas, due to their vulnerability.

Nature Park Golija is one of the most important protected natural areas in Serbia, considering its size, geodiversity, biodiversity, and the fact that its largest part is the UNESCO MaB Reserve Golija-Studenica. According to the Forest Fire Susceptibility Index method, 24.71% of the nature park territory is considered to be in the zones of very high and high forest fire susceptibility, and according to the fuzzy AHP method, that share is 23.68%. Those figures are very unfavorable; therefore, in areas that are considered to be of higher susceptibility, it is necessary to intensify the implementation of measures in order to reduce forest fire risk. When considering forest fire susceptibility in the MaB reserve, zones of very high and high forest fire susceptibility are recorded on 26.85% (Forest Fire Susceptibility Index) and 25.75% (fuzzy AHP).

The design and deployment of the proposed IoT-based sensor network and the overall system that encompasses real-time measurements, forest fire risk prediction, and fire outbreak detection present a cost-effective, reliable, and flexible long-term solution for the collection of meteorological data and data on fire outbreak occurrence, and for the improved short-term and long-term forest fire susceptibility assessment, which will certainly enable a more advanced forest fire prediction, detection, and monitoring activities. Additionally, due to the constant collection of all-important data, this system could be continuously improved through the usage of more advanced inference techniques (i.e., machine learning and deep learning), without substantial investments.

Obtained results can be applied for adequate forest fire risk management, improvement of the monitoring, and an early warning system in the Republic of Serbia. The forest fire susceptibility maps and spatial data obtained by research are of the utmost importance for decision makers for better preparedness, coordination, and intervention in the case of forest fire occurrence. The lessons learned from the study area will help scale up the forest fire susceptibility assessment on the territory of the whole country, and especially in protected natural areas.

## Figures and Tables

**Table 1:** Values of classes in factors.

Class Value	Vt	S [°]	A [°]	Ds [m]	Dr [m]	Df [m]
5	Coniferous forest	>35	S (157.5–202.5)	<500	<150	<200
4	Mixed forest; shrub	25–35	SE (112.5–157.5); SW (202.5–247.5)	500–1000	150–300	200–400
3	Broad-leaved forest	10–25	E (67.5–112.5); W (247.5–292.5)	1000–1500	300–450	400–600
2	Agricultural areas; grassland	5–10	NE (22.5–67.5); NW (292.5–337.5)	1500–2000	450–600	600–800
1	Artificial areas; barren rocks	<5	N (0–22.5, 337.5–360)	>2000	>600	>800

**Table 2:** Forest fire susceptibility zones classification according to performance index values.

Performance Index Value	Forest Fire Susceptibility Zone
0.0–0.2	Very high
0.2–0.4	High
0.4–0.6	Medium
0.6–0.8	Low
0.8–1.0	Very low

**Table 3:** Fuzzy sets and fuzzy domains proposed for linguistic variables.

Variable	Fuzzy Set	Fuzzy Domain
Temperature (T)	FS_T_ = {low, medium, high, extreme}	[0, 100] °C
Humidity (H)	FS_H_ = {low, medium, high, extreme}	[0, 100] %
Wind speed (W_speed_)	FS_W_ = {low, medium, high, extreme}	[0, 240] km/h
Rainfall (R)	FS_R_ = {low, medium, high, extreme}	[0, 100] mm/m^3^
Oxygen (O_2_)	FS_O2_ = {low, medium, high, extreme}	[0, 30] %
Carbon dioxide (CO_2_)	FS_CO2_ = {low, medium, high, extreme}	[0, 1000] ppm
Carbon monoxide (CO_2_)	FS_CO_ = {low, medium, high, extreme}	[0, 100] ppm

**Table 4:** Measurement frequency and average calculation of fire risk factors.

Forest Fire Risk	Fire Outbreak Occurrence	Measurement Frequency	Average Calculation
Non-existent	Non-existent	5 minutes	Last 20 measurements
Low	/	2 minutes	Last 15 measurements
High	/	Continuous	Last 10 measurements
Extreme	5–10	Continuous	Last 5 measurements

**Table 5:** Distribution of classes in factors in the total Nature Park Golija territory.

Class Value	Vt [%]	S [%]	A [%]	Ds [%]	Dr [%]	Df [%]
5	9.44	1.32	10.31	66.98	71.50	62.74
4	22.25	13.21	23.94	26.47	22.69	18.54
3	48.47	66.79	28.23	5.68	4.86	9.69
2	17.91	13.87	25.91	0.86	0.82	4.93
1	1.93	4.81	11.61	0.01	0.12	4.11

**Table 6:** Fuzzy comparison pairwise matrix.

	Vt	A	S	Dr	Ds	Df
**Vt**	1, 1, 1	1, 2, 3	1.5, 2.5, 3.5	2, 3, 4	2, 3, 4	2, 3, 4
**A**	0.33, 0.5, 1	1, 1, 1	1, 2, 3	1, 2, 3	1, 2, 3	1, 2, 3
**S**	0.29, 0.4, 0.67	0.33, 0.5, 1	1, 1, 1	1, 2, 3	1, 2, 3	1, 2, 3
**Dr**	0.25, 0.33, 0.5	0.33, 0.5, 1	0.33, 0.5, 1	1, 1, 1	1, 1, 1	1, 2, 3
**Ds**	0.25, 0.33, 0.5	0.33, 0.5, 1	0.33, 0.5, 1	1, 1, 1	1, 1, 1	1, 2, 3
**Df**	0.25, 0.33, 0.5	0.33, 0.5, 1	0.33, 0.5, 1	0.33, 0.5, 1	0.33, 0.5, 1	1, 1, 1

**Table 7:** Weighted values (w_j_) of factors obtained from the fuzzy AHP method.

Factors	w_j_
**Vt**	0.311
**A**	0.233
**S**	0.196
**Dr**	0.108
**Ds**	0.108
**Df**	0.044

**Table 8:** Share of forest fire susceptibility zones in total nature park area.

Susceptibility Class	RC [%]	Fuzzy AHP [%]
Very high	0.48	0.49
High	24.23	23.19
Medium	70.51	69.05
Low	4.78	7.26
Very low	0.0002	0.01

**Table 9:** Share of forest fire susceptibility zones in total area of different regimes of protection.

Susceptibility Class	RC [%]			Fuzzy AHP[%]		
	I	II	III	I	II	III
Very high	0.11	0.70	0.47	0.11	0.69	0.48
High	32.44	34.17	23.49	32.04	34.11	22.38
Medium	66.22	63.01	71.05	65.59	61.80	69.56
Low	1.23	2.12	4.99	2.27	3.39	7.57
Very low	0.00	0.00	0.0002	0.00	0.01	0.01

**Table 10:** Share of forest fire susceptibility zones in total MaB reserve area.

Susceptibility Class	RC [%]	Fuzzy AHP [%]
Very high	0.57	0.59
High	26.28	25.15
Medium	69.17	68.04
Low	3.97	6.21
Very low	0.00	0.003

## Data Availability

The data presented in this study are available on request from the corresponding author.
